# Capturing Community Perspectives in a Statewide Cancer Needs Assessment: Online Focus Group Study

**DOI:** 10.2196/63717

**Published:** 2025-07-31

**Authors:** Jessica R Thompson, Keeghan Francis, Caree R McAfee, Madeline Brown, Todd Burus, Melinda Rogers, Connie L Sorrell, Elizabeth Westbrook, Lovoria B Williams, Jennifer Redmond Knight, Elaine Russell, Natalie P Wilhite, Pamela C Hull

**Affiliations:** 1 Department of Health Policy and Administration The Pennsylvania State University University Park, PA United States; 2 Community Impact Office University of Kentucky Markey Cancer Center Lexington, KY United States; 3 Kentucky Cancer Program-East University of Kentucky Markey Cancer Center Lexington, KY United States; 4 Kentucky Cancer Program-West University of Louisville Louisville, KY United States; 5 College of Nursing University of Kentucky Lexington, KY United States; 6 Department of Health Management and Policy University of Kentucky Lexington, KY United States; 7 Kentucky Cancer Consortium University of Kentucky Markey Cancer Center Lexington, KY United States; 8 Department of Behavioral Science University of Kentucky Lexington, KY United States

**Keywords:** cancer, needs assessment, focus groups, virtual data collection, qualitative research, health equity

## Abstract

**Background:**

Kentucky has the highest all-site cancer incidence and mortality rates in the United States. Conducting needs assessments in a large geographic area, such as an entire state, poses challenges in collecting qualitative data from diverse rural and urban contexts. In 2021, a steering committee was formed to drive a multimethod, statewide cancer needs assessment (CNA) to identify the future priorities for all cancer-related care in Kentucky.

**Objective:**

We aimed to report on the online focus group component of the CNA by documenting existing community resources and perceived needs across the cancer care continuum. In addition, we aimed to explore the impacts of social determinants of health among populations experiencing health disparities.

**Methods:**

Through existing partnerships and a national research registry, we recruited adult Kentucky residents who were not employed in health occupations to participate in 11 online 60-minute focus groups, stratified to include multiple target populations and geographic areas. We based our semistructured discussion guide on the cancer care continuum and focused on social determinants of health, health equity, and factors affecting cancer diagnoses and outcomes. We conducted a qualitative line-by-line analysis of the recorded transcripts to identify themes.

**Results:**

The participants (N=51; mean 4.63, SD 2.26 per group) lived in 25 different counties, including 35% (18/51) from rural communities, 14% (7/51) from the Appalachian area of Kentucky, and 31% (16/51) who self-identified with a racial or ethnic minority group. We identified 17 primary themes representing community-perceived needs and potential solutions across the cancer care continuum, including novel approaches to make information accessible; messaging not interpreted as blaming or shaming; messaging from individuals who engender trust; screening efforts to reach individuals where they are; ways to address practical barriers to screening and treatment, such as cost and transportation; and ways to increase knowledge about insurance coverage. In addition, we found 83 emergent subthemes specific to race, ethnicity, rural and urban residence, sexual orientation and gender identity, and age. The participants described the need to promote positive, culturally sensitive patient–health care provider communication and to create safe care spaces that consider the ways in which social norms affect cancer care, fight stigma, and improve health equity.

**Conclusions:**

By conducting statewide qualitative data collection online, we provided valuable depth of understanding for future programs and research to address cancer incidence and mortality in Kentucky. The findings pointed to several potential actions to address community-perceived needs across the cancer care continuum, including increasing accessible risk reduction information, expanding ways to overcome challenges to screening and treatment, building patient navigation resources, and increasing positive patient–health care provider communication. The findings also suggest that online focus groups can be a valuable component of CNAs to capture cancer-related needs and solutions across large geographic areas and diverse populations.

## Introduction

### Background

The Commonwealth of Kentucky has the highest all-site cancer incidence and mortality rates in the United States [1]. In 2021, a group of academic and community partners collaborated on a comprehensive, statewide cancer needs assessment (CNA) to address the state’s extensive cancer burden. The use of CNAs has increased in recent decades, grounded in their promotion by the Affordable Care Act and accreditation boards, as well as requirements by the National Cancer Institute (NCI) for NCI-designated cancer centers to assess the needs of their catchment areas. In this case, the catchment area of focus consisted of the entire state of Kentucky. In this CNA, we sought to explore the lived experiences of community members through the examination of specific topics across the cancer care continuum, from risk reduction to treatment follow-up, through online focus groups. This approach allowed us to reach individuals throughout the diverse geographic regions and cultural contexts of the state, encompassing multiple urban areas and a large rural population, which includes the predominantly rural Appalachian region.

In CNAs and other community health needs assessments, qualitative methods can complement quantitative data to identify and shed additional light on the needs and inequities, such as those by race, ethnicity, and geography, in services across the cancer care continuum [2-5]. Qualitative methods provide an ideal way to understand the depth and richness of experiences behind the patterns and trends noted in quantitative data [2,6] and to include community perspectives in the prioritization of needs [4,7]. Speaking directly with community members can highlight their varying perspectives compared to those of health care providers. For example, health care providers often focus clinically on cancer needs, whereas community members more regularly focus on ways to address relevant social barriers and health disparities [6,8].

Previous qualitative research suggests that community member perspectives about needs across the cancer care continuum may vary across groups [5]. For example, rural and urban residents may obtain information about cancer risk reduction from different sources; rural individuals may be more exposed to misleading information around risk reduction and screening due to reduced access to the internet or health care providers capable of verifying accurate information [9]. Individuals in underserved populations (eg, those with limited access to resources, services, or health care) also display variation in the amount of knowledge they possess about when, where, and how to secure treatment, and many may have trouble accessing care because of distance or clinic hours [3]. Similarly, members of racial and ethnic minority groups desire culturally appropriate educational materials about screening and treatment specific to their communities [3,6]. Previous studies [3,10] have identified unique needs for cancer support or support programs among rural populations and racial and minority groups, such as concerns about paying for services and an increased need for survivorship services, including community-specific support groups. Among populations who experience cancer-related health inequities, previous studies [2,3,6] have indicated a wide range of factors, including environment (eg, political and physical), discrimination, stigma, distrust, and strategies to improve access to health care (eg, geographic distance and costs).

Traditionally, qualitative focus groups have been conducted in person, yet technological advances within the past decade have made it possible to conduct online groups using videoconferencing [11,12]. Although the use of online focus groups in health research was relatively uncommon before 2020, the COVID-19 pandemic stimulated a rapid increase in the uptake of videoconferencing technology for work, education, telehealth, and personal uses [13]. The pandemic restrictions on in-person contact caused disruptions to data collection activities, prompting many researchers to explore online data collection options, such as online focus groups [12]. Some studies [11,14] have reported successfully using online focus groups during the pandemic with hard-to-reach populations and for uncovering cancer-related needs, such as colorectal [15] and breast cancer screening [16]. Although technological limitations can occur with online administration, promising advantages also exist, including reduced travel barriers for participants who live further away, increased comfort from the ability to participate at home or other convenient locations, and increased flexibility of scheduling. Online focus groups have demonstrated the ability to gather data on par with in-person focus groups while maintaining the pandemic safety protocols, allowing research teams to gather important and timely data [11,14].

### Objectives

To the best of our knowledge, this is the first use of online focus groups as a part of a statewide cancer-focused CNA. Specifically, the objectives of the CNA focus group component were (1) to document existing community resources and needs across the cancer care continuum domains (risk reduction, screening, diagnosis, treatment, follow-up, and survivorship) [5] and (2) to explore ways that the social determinants of health affect cancer care throughout Kentucky and the unique needs of groups that experience cancer health disparities based on race, ethnicity, geographic area, sexual orientation, or gender identity.

## Methods

### Kentucky CNA and Community-Engagement Overview

In 2021, the Community Impact Office (CIO) in the University of Kentucky Markey Cancer Center (UKMCC) convened a steering committee to collaborate on a new CNA for Kentucky. The 27 steering committee members represented diverse organizational partners with statewide reach and interest in cancer, including staff of the Kentucky Cancer Consortium (KCC), the Kentucky Cancer Program (KCP), the Kentucky Cancer Registry, the American Cancer Society, the Foundation for Healthy Kentucky, the Kentucky Department for Public Health, and the University of Louisville. The overall CNA used a mixed methods approach, which is described in more details elsewhere [17]. Quantitative data were compiled from state and national data sources [18] (eg, the US Census Bureau’s American Community Survey, the Kentucky Cancer Registry as part of the NCI’s Surveillance, Epidemiology, and End Results Program, and the Centers for Disease Control and Prevention’s Behavioral Risk Factor Surveillance System [19-21]) to describe patterns in key indicators and areas of cancer disparities in Kentucky. To complement the quantitative data, the CNA design included gathering qualitative data to better understand the experiences, perceptions, and needs of Kentuckians.

The CNA steering committee, including the KCP and the KCC members, gave valuable feedback on the focus group sampling design, recruitment strategies, discussion guide questions, and incorporation of the findings into the overall CNA. The steering committee used the CNA conceptual framework, the development of which is described elsewhere [17], to create the focus group discussion guide. Coauthors of this publication wrote the questions, followed by the review and revision by the steering committee members, who included individuals with relevant expertise and partner organizations. The KCP is co-led by the UKMCC CIO and the University of Louisville Brown Cancer Center. Founded in 1982 by the state legislature, the KCP is a statewide community-based cancer prevention and control program served by regionally located staff covering each of Kentucky’s 15 area development districts. The regional KCP staff manage District Cancer Councils comprising local and regional organization partners, such as health providers, faith-based leaders, cancer survivors, and educators. The KCP staff plan and implement evidence-based programs, in collaboration with these and other partner organizations, focused on cancer risk reduction, early detection, and survivorship. The KCC, created in 2002 and funded by the Centers for Disease Control and Prevention Comprehensive Cancer Control Program, is the state’s comprehensive cancer control coalition and is managed by staff in the UKMCC CIO. The KCC convenes a wide range of statewide partner organizations, who collectively develop, implement, and evaluate the state’s cancer plan with particular focus on health equity and policy, systems, and environmental changes. The KCP and the KCC staff members contributed to the focus group question guide, distributed the focus group recruitment flyer throughout their partner networks, sat in on the focus group discussions, and provided feedback in the postgroup debrief discussions with facilitators.

### Study Population and Recruitment

For inclusion, participants had to be aged ≥18 years and a resident of Kentucky. Because our target population was lay community members, we excluded individuals who reported working in public health, health care, or another health-focused position. If participants voiced a lack of experience with Zoom (Zoom Communications, Inc), we provided one-on-one training to ensure comfort with the online setting. We excluded participants who reported not feeling comfortable participating in an online focus group via Zoom and declined our one-on-one support [22]; less than 5% (2/51) of the participants expressed concerns over participation in this format, all of whom received one-on-one support.

Through the KCC and the KCP partnership networks, we distributed flyers that linked participants to an eligibility screener in REDCap (Research Electronic Data Capture; Vanderbilt University) [23]. The KCC develops and distributes a weekly e-newsletter to more than 600 subscribers. The flyer was included in the newsletter over multiple weeks. The KCP members distributed flyers to their existing email lists of partners throughout Kentucky and posted printed flyers at public community events. We also used ResearchMatch, a national registry created by several academic institutions and supported by the US National Institutes of Health as part of the Clinical Translational Science Award program, to identify potential participants [24]. Within ResearchMatch, we developed a search for adult residents in Kentucky. ResearchMatch allows for the direct distribution of recruitment materials and eligibility screener link to potential participants in the search results.

To increase heterogeneity and diversity, we used a stratified purposive sampling strategy based on race and ethnicity; geography (rural and urban, as well as Eastern and Western Kentucky, to capture varying cultural contexts); and sexual orientation or gender identity, with intentional oversampling of Black and LGBTQ+ (lesbian, gay, bisexual, transgender, queer, and other minority) individuals. We determined rural-urban designations at the county level from the 2013 rural-urban continuum codes [25]. We gathered the aforementioned demographics in the eligibility screener and assigned participants to focus groups based on these characteristics and preferences for group participation. The recruitment continued until we had enough participants to schedule each of the following groups: (1) urban eastern Kentucky (mixed race or ethnicity), (2) urban eastern Kentucky (Black or African American), (3) urban western Kentucky (mixed race or ethnicity), (4) urban western Kentucky (Black or African American), (5) rural western Kentucky, (6) rural eastern or Appalachian Kentucky, (7) Hispanic or Latine (any location), and (8) LGTBQ+ (any location). We recruited enough participants in a few of these groups to warrant multiple focus group sessions.

### Ethical Considerations

The study team contacted eligible participants based on identified preference (phone, text, or email) to provide consent information via a cover letter, schedule the focus group, and provide a link to a brief demographic questionnaire in REDCap. All participants received consent information via email when starting the demographic questionnaire, as well as by oral review before starting the focus group. The consent explanation included a description of actions taken to ensure privacy, including the removal of any identifying information in the dataset and the deletion of recordings once completing analysis. We proceeded with data collection once receiving verbal consent from all participants in each group. Participants received a US $50 e-gift card for participation. We received a waiver to document consent for this project, and all procedures were approved by the University of Kentucky Institutional Review Board as an expedited study (65451).

### Data Collection

**Figure 1:**
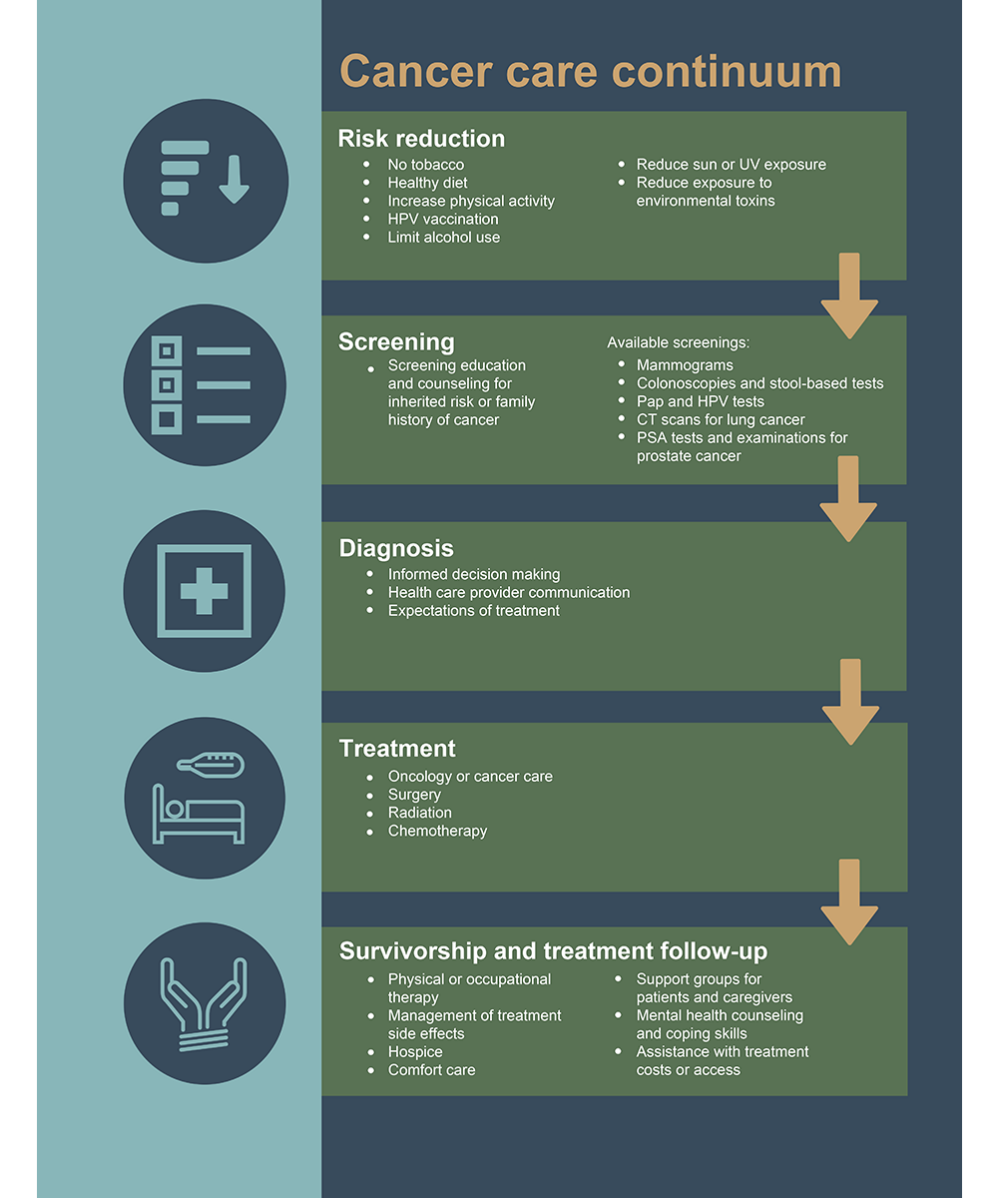
Graphic representation of the cancer care continuum displayed during the focus groups (informed by the Institute of Medicine, 2013 [5]). CT: computed tomography; HPV: human papillomavirus; PSA: prostate-specific antigen.

From July to September 2021, we conducted 11 focus groups. A trained facilitator (JRT) and a research assistant and notetaker (KF) conducted the 2-hour sessions on Zoom. Before proceeding with the semistructured discussion guide questions (Multimedia Appendix 1) to introduce the cancer care continuum domains [5] and allow the participants to ask clarifying questions, the facilitator used Zoom’s screen sharing feature to show a visual graphic of the relevant factors for risk reduction, screening, diagnosis, treatment, treatment follow-up, and survivorship (Figure 1). One continuum domain was discussed at a time. To discuss risk reduction, we highlighted various health behaviors and exposures that increase cancer risk, including tobacco use, diet, physical activity, sun exposure, and exposure to environmental toxins. For the screening discussion, we provided participants with examples of available screening types, including mammography for breast cancer, Papanicolaou and human papillomavirus tests for cervical cancer, colonoscopies and stool-based tests for colon cancer, low-dose computerized tomography scans for lung cancer, prostate-specific antigen tests and examinations for prostate cancer, and genetic testing for inherited risk. To set the stage for discussing diagnosis and treatment, we expressed our interest to the participants in understanding more about conversations that occur at the point of diagnosis and the types of treatment available in their communities, such as surgery, radiology, and chemotherapy. In our discussion of cancer treatment follow-up and survivorship, we covered an array of potential resources, such as support groups, physical and occupational therapy, financial supports, mental health supports, and palliative care, as well as side effects from treatment. For each continuum area, the questions focused on participant awareness of local resources and the challenges that the individuals faced in their community related to each domain. Through the intentional scheduling of participants in the focus groups with people sharing similar demographic characteristics, we also sought to identify emergent themes and compare them across groups.

### Analysis

We audio- and video-recorded each session and transcribed the resulting recordings verbatim. The facilitator (JRT) and research assistant (KF) double-coded 20% of the transcripts in Microsoft Excel with >90% initial agreement. To ensure coding alignment and analysis rigor, we resolved discrepancies through discussion and consultation with a third author (PCH). All 3 authors identify as female and have experience in public health and qualitative research; two of the authors (JRT and PCH) have doctoral degrees in health and extensive expertise in community-engaged approaches, including with low-income, rural, and minority populations. For coding, we combined two approaches: (1) a deductive approach based on the semistructured focus group questions to isolate the specific resources and needs across the cancer care continuum and (2) an inductive approach to identify emergent themes from demographic groups who experience cancer health disparities related to race and ethnicity, geographic location, and sexual orientation or gender identity [26,27]. The deductive approach resulted in 13 primary themes across the continuum of care, along with the considerations related to the COVID-19 pandemic. We conducted the focus groups until thematic saturation was reached within these 13 primary themes in order to provide sufficient feedback in each of the cancer care continuum domains. The subsequent inductive approach resulted in 83 emergent subcodes, including specific differences across the demographic groups experiencing health disparities.

## Results

### Participant Characteristics

A total of 51 adults from 25 of Kentucky’s 120 counties participated in the 11 focus groups. We scheduled 79 participants, resulting in an attendance rate of 65% (51/79). The mean focus group size was 4.63 (SD 2.26) participants. The sample had a mean age of 49 years. Among the 51 participants, 18 (35%) were from rural communities, 7 (14%) were from Appalachian Kentucky, and 16 (31%) self-identified as Black, American Indian, Asian, or Hispanic or Latino. Table 1 provides a summary of the participant demographic characteristics. Although the stratified purposive sampling design was not intended to yield a sample that is directly representative of the Kentucky population, the participant demographics were similar to the state, with intentional oversampling of Black and LGBTQ+ individuals.

**Table 1:** Demographics of the cancer needs assessment focus group participants (N=51) compared to state-level demographics (N=4,449,052).

Demographic variables		Sample	State of Kentucky
Age (y), median		49.0	39.1
**Sex or gender identity, n (%)**			
	Female	40 (78.4)	2,258,130 (50.8)
	Male	11 (21.6)	2,190,922 (49.2)
	Nonbinary	0 (0)	—^a^
**Rural or urban residency^b^, n (%)**			
	Urban (metro) county	33 (64.7)	2,619,409 (58.9)
	Rural (nonmetro) county	18 (35.3)	1,829,643 (41.1)
Appalachian County		7 (13.7)	1,165,722 (26.2)
**Race and ethnicity^c^, n (%)**			
	American Indian	2 (3.9)	9386 (0.2)
	Asian	2 (3.9)	65,191 (1.5)
	Black	11 (21.6)	358,928 (8.1)
	Hispanic or Latino	1 (2)	162,994 (3.7)
	White	35 (68.6)	3,868,479 (87)
LGBTQ+^d^ identity		4 (7.8)	—

^a^Not available.

^b^Determined by the county-level rural-urban continuum codes; a code ≥4 represents a rural county.

^c^Responses are not mutually exclusive because the participants could choose more than one.

^d^LGBTQ+: lesbian, gay, bisexual, transgender, queer, and other minority.

### Major Themes Across the Cancer Care Continuum

#### Overview

**Figure 2:**
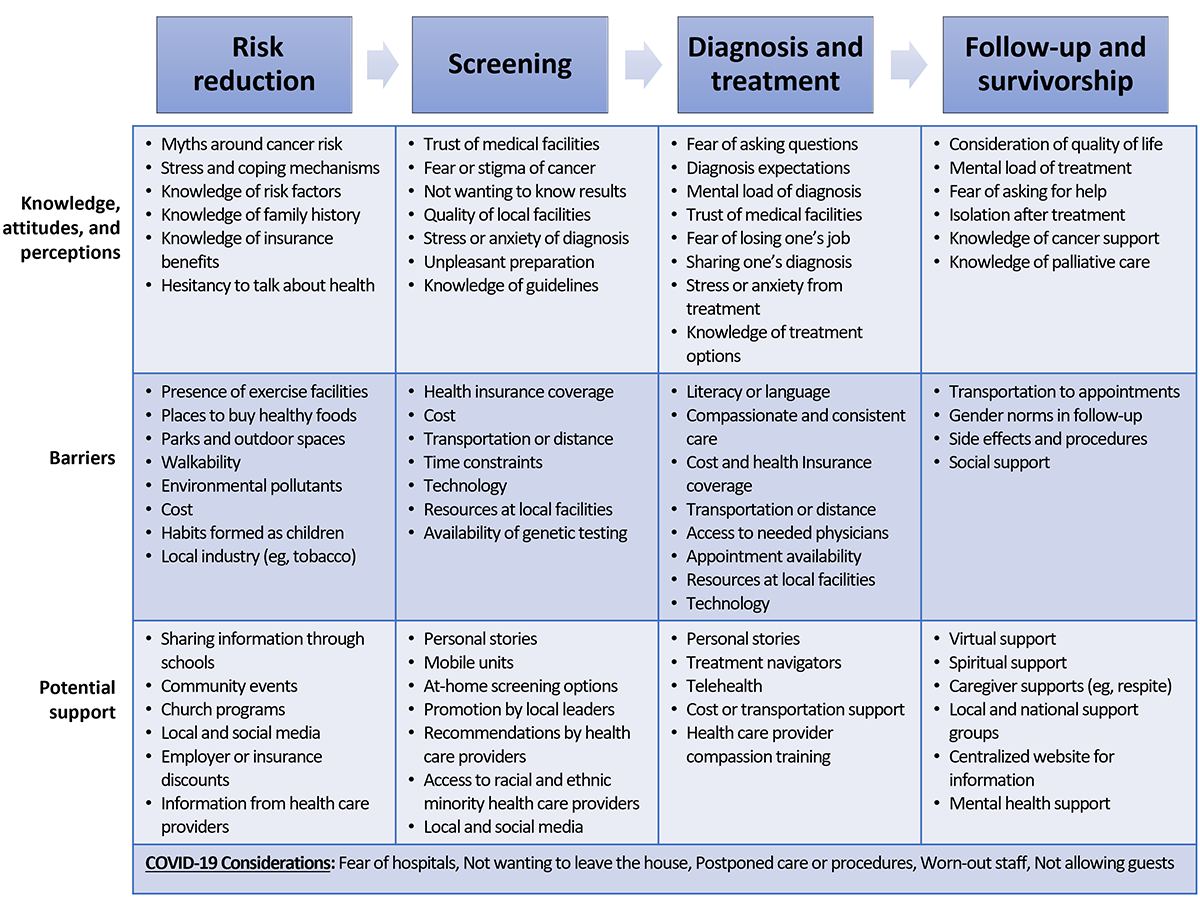
Summary of the themes and subcodes of the cancer needs assessment focus group categorized by the cancer care continuum.

In reaction to the cancer care continuum graphic (Figure 1), participants described the needs and associated potential solutions for each domain of the continuum—risk reduction, screening, diagnosis, treatment, treatment follow-up, and survivorship. Refer to Figure 2 for a list of the 83 identified subcodes within each of the 13 primary themes, which are organized into the 12 blocks within the table, along with the considerations of the COVID-19 pandemic that apply to the entire continuum of care. The identified representative quotes for each continuum domain are provided in Table 2.

**Table 2:** Representative quotes by cancer care continuum area.

Continuum area	Representative quotes
Risk reduction	“There’s a lot of folks that don’t live in the downtown area or a walkable part of our town that will never make it 5 miles to the [University of Kentucky Cooperative] Extension Office [or] don’t know that the Extension Offices have fabulous nutritional programming.” [White woman from a rural county; aged 31 y] “My husband still smokes heavily, and he knows all the risk and he still does it because he’s addicted to it. I think that preventing it from happening [in the first place] is definitely a concern. I know in my area, my dad and mama did, their uncle did, their grandpa did. [When I] ask, they say, ‘Well, I started smoking at seven when I got a cigarette from my grandpa,’ and it's just old family mentality circles.” [Asian woman from a rural county; aged 31 y] “We could definitely have a bigger presence [of environmental pollutant awareness] in my specific area, especially with how much agriculture we have...the runoff can cause a lot more issues than I think most of our residents are aware of.” [White woman from a rural county; aged 32 y] “It’s about people feeling attacked, so I think it just really goes back to messaging, and I think...people always want to know why, like what’s your motivation for doing it, so I think the better you can communicate that.” [White woman from an urban county; aged 36 y]
Screening	“Well, you know the colon [screening], it’s not so much that the procedure’s horrible but the process of getting ready for the procedure is not pleasant, and I know that I’m aware of that. And even though my best friend has gone through the process, she shared it, so I’m trying to hold off.” [Black woman from an urban county; aged 45 y] “I can remember when they cut back on pap smears I was shocked. I don’t see the benefit, and, okay, now I only need one every 3 years, and I’m like, that doesn't seem to make sense to me.” [White woman from an urban county; aged 36 y] “Another issue is that even people that have insurance and then all the things [she] said, like people that work all the time that are working two jobs or they don’t have someone to watch their kids. They don’t have transportation. That’s a big problem here, we don’t have public transit.” [White woman from an urban county; aged 44 y; LGBTQ+^a^ identity] “I think it was mammogram screening...the mobile clinic, so I think that’s a really good idea that they came up with, and you know, it’s able to go around and meet the people where they are.” [Black woman from an urban county; aged 48 y] “Well, I think even people that have insurance, the deductibles are so high on a lot of them that there are people that will not [get screened]. I mean there’s a few things on this list that are now because of the ACA [Affordable Care Act] you can get screened once a year, and you don't have to pay for it [if you know about it].” [White woman from a rural county; aged 45 y]
Diagnosis and treatment	“These [radiation and chemotherapy] are available in my community, but most people that have means don’t want to receive them here...A lot of people that have means would go elsewhere. They would go to [hospital in nearby city] or somewhere else. Or, I know people that may go there [locally] the first time, and then when they get diagnosed, if they have the means they go to [hospitals in larger nearby cities].” [White woman from a rural county; aged 44 y; LGBTQ+ identity] “The ease of medical information being shared...would help with treatment, knowing all your doctors are on the same page...knowing what each person, what the right hand is doing and what the left hand is doing at the same time.” [White woman from an urban county; aged 45 y] “The way that the health care system is set up right now takes too long. So, if you go in and find a lump or whatever, its 2 weeks or a month before you go to the next step and then it’s even more time after that, before you know what stage it is, and then a treatment plan. That is a lot of heavy mental load for somebody to have to carry for that long.” [Black woman from an urban county; aged 48 y] “I think a lot of times, you have to recognize when you need a proxy or for the health care staff to recognize when someone needs a proxy because you’re saying something to somebody and saying, ‘do you understand?’ They're going to say ‘yes’ because they don’t want to appear that they don’t know, but just being able to understand and to ask the type of questions they need to.” [Black woman from an urban county; aged 28 y]
Follow-up and survivorship	“I think they gave her family false hope and did things that work for some people because they’re younger...but probably lowered her quality of life at the end when it was inevitable that she was going to pass away.” [White woman from an urban county; aged 73 y; LGBTQ+ identity] “They found that she had Medicaid it’s like ‘oh we’re sorry, this is an experimental treatment, and this isn’t covered by Medicaid.’ So, [we asked] how much does this cost. It will cost you about $100,000, and it might as well have been a million dollars. I have followed the research and it has been phenomenal, and it’s so sad that we live in a world that $100,000 is worth more than an extra 10 to 15 years of my mother’s life.” [White man from an urban county; aged 41 y; LGBTQ+ identity] “And the other person I met with...was like ‘well, I don’t understand why a young, pretty women like you wouldn’t...want to have that surgery done.’ So, I think that there is sort of pressure to meet traditional gender stereotypes in reconstruction, and you know, like how you deal with that that sort of thing, and also with like fertility issues.” [White woman from a rural county; aged 45 y] “We probably need more mental health professionals down here. When I first got a cancer diagnosis, the insurance company sent me a letter in the mail that I could talk to an oncology nurse, and I talked with her and that was really helpful to have like a third-party person not involved in like my achieving my plan and not family or friend who has any emotional investment but also not my doctor to talk to. That was a really great support.” [White woman from an urban county; aged 74 y]

^a^LGBTQ+: lesbian, gay, bisexual, transgender, queer, and other minority.

#### Risk Reduction

##### Knowledge, Attitudes, and Perceptions

The participants identified several knowledge, attitude, and perception-based factors that affect cancer risk reduction behaviors. The factors included myths around cancer risk; knowledge of risk factors, family history, and insurance benefits; stress and related coping mechanisms; and a hesitancy to talk about health or cancer.

##### Barriers

The participants also described various barriers to risk-reduction behaviors. For example, participants cited the absence or lack of access to places to buy healthy foods, exercise facilities, parks, and outdoor spaces, as well as walkable neighborhoods, as adversely affecting their diet and physical activity. Walkability and proximity also affected access to community risk reduction resources, such as tobacco cessation, radon testing, and other resources found at local health departments or extension offices. Participants also perceived family history and historical livelihoods as barriers to cancer risk reduction, including health habits formed as children (eg, diet and exercise) and generational influences (eg, tobacco use). Similarly, participants expressed a lack of awareness around the role of local industry (eg, tobacco farming, mining, and factories) in creating potential environmental pollutants. Finally, participants identified cost as a major barrier to healthy eating and physical activity.

##### Potential Solutions

When discussing potential solutions to these challenges, the participants described strategies to increase education and knowledge of resources to support risk-reduction behaviors, including several modes to share information. Participants named community events (eg, health fairs and local festivals), schools, churches, local and social media, local major employers, insurance companies, and health care providers as possible strategies to convey information and increase knowledge around cancer risk reduction in Kentucky communities. In addition, participants emphasized that information and messaging should be free from shame or blame language, using positive and constructive suggestions.

#### Screening

##### Knowledge, Attitudes, and Perceptions

The participants described a variety of knowledge, attitudes, and perceptions affecting the receipt of screening, falling into two major categories: (1) perceptions of medical facilities and (2) personal concerns or knowledge of screening. The former included aspects such as trust in medical facilities and health care providers, and the quality of screening resources available at local facilities. The latter included aspects such as fear or stigma surrounding cancer, stress or anxiety of diagnosis, unpleasant preparation, knowledge of screening guidelines, and not wanting to know the results due to the cost, stress, and other next steps related to a potential cancer diagnosis. For example, participants described the effects of unpleasant colon screening preparation and the confusion that individuals may feel in light of changing screening guidelines.

##### Barriers

The participants identified practical challenges in receiving cancer screening. The most commonly raised barriers were cost, health insurance coverage, and transportation or distance to facilities. In addition, participants mentioned time constraints (eg, clinic hours, ability to take time off work, and childcare responsibilities); technology barriers; and availability of local resources for screening and genetic testing.

##### Potential Solutions

As solutions for screening challenges, the participant recommendations fell into two areas: (1) increasing access by reaching people where they are and (2) ways to boost or promote screening messaging. Increasing access included the use of mobile units and at-home screening options (eg, stool-based tests for colon cancer). Participants suggested that screening messaging could have a greater impact if it came from local leaders, trusted local health care providers, providers of similar racial or ethnic minority background and gender identity, and personal stories from cancer survivors. As with risk reduction, participants recommended that screening messaging or opportunities could be effectively implemented or shared through community events, schools, churches, local and social media, and insurance companies. Participants recommended developing strategies to increase knowledge of which preventive screenings are covered by insurance, as well as their eligibility and frequency.

#### Diagnosis and Treatment

##### Knowledge, Attitudes, and Perceptions

At the point of diagnosis, the participants indicated that individuals experience a large mental load and various associated effects. Discussion of the diagnosis mental load included stress, anxiety, fear of losing one’s job, fear of asking questions, and understanding diagnosis expectations. Participants also expressed a need for skills around discussing one’s cancer diagnosis with family and friends and to gain self-efficacy in order to make confident treatment choices, ask health care providers questions, or receive second opinions. As with screening, participants described that their trust in medical facilities and health care providers, as well as their perceptions of the quality of treatment resources at local facilities, affected their treatment decisions.

##### Barriers

The participants discussed a wide variety of communication challenges with health care providers related to diagnosis and treatment decisions. For example, they described the importance of literacy and language barriers; compassion and understanding among health care providers; and challenges with consulting multiple specialists, including the sharing of information among these physicians. As with screening, participants named several logistical and practical barriers, such as cost, health insurance coverage, transportation and distance to health facilities that provide the needed treatments, appointment availability, and technology barriers to telehealth.

##### Potential Solutions

For potential solutions, the participants discussed the importance of navigators in the diagnosis experience to offset the mental load and to assist in explaining prognosis and treatment options. Similarly, social supports and personal stories from friends and family were key to diagnosis and treatment decision-making and appointment keeping. Participants also requested more information on cost or transportation supports, as well as telehealth options, for those with technological access and skills to complete these visits. Finally, to improve patient–health care provider communication, participants recommended increased training in compassion and humility for health care providers.

#### Treatment Follow-Up and Survivorship

##### Knowledge, Attitudes, and Perceptions

The participant perceptions included the continued mental load during treatment, isolation after treatment, fear of asking for continued help, and considerations of the quality of life during treatment decisions. In addition, participants described the need for knowledge about cancer support, support programs, and palliative care, particularly before the patient or family needs them.

##### Barriers

Transportation to health facilities and the cost of treatment continued to be a challenge during this period. Participants expressed the need for social supports and the complexities of receiving referrals for therapy or other supports. In addition, participants discussed the side effects from procedures and medications and the way social norms around gender and age affect health care provider and community perspectives on treatment follow-up choices, such as breast reconstruction. Follow-up support or support programs varied based on location, age, and cancer diagnosis, where some had plentiful and others had few resources available for their diagnosis or age group.

##### Potential Solutions

The participants described the continued need for survivor supports, including through local and national support groups. Participants suggested the use of online support groups, as well as those at faith-based locations, to increase access. In addition, participants discussed the importance of caregiver supports, such as respite, and for reducing stigma around mental health, along with increasing access and knowledge of these forms of support. Finally, participants requested a single location, such as a centralized website, to find information on available treatments and supports.

### Social Determinants of Health and Health Equity: Population Differences

#### Overview

**Figure 3:**
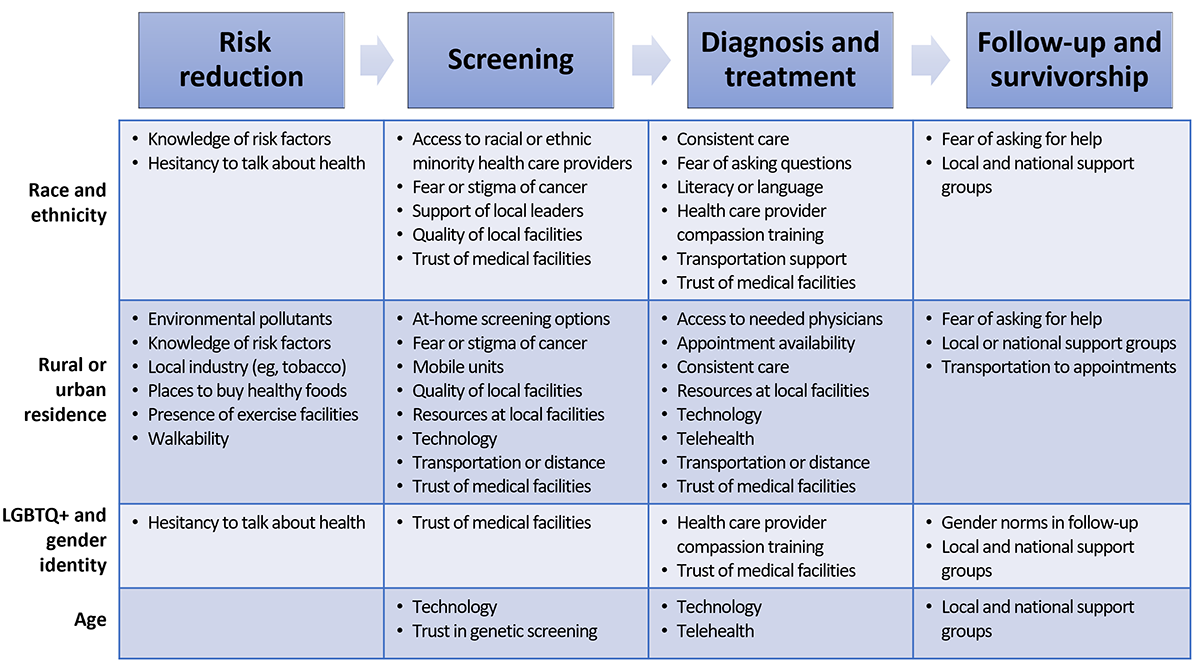
Summary of the emergent themes across the cancer care continuum, specifically raised among population groups who experience cancer health disparities. LGBTQ+: lesbian, gay, bisexual, transgender, queer, and other minority.

We found several specific differences between the groups that elucidated the ways in which social determinants of health affect populations across the cancer care continuum. Figure 3 includes the emergent themes across the continuum of care that were particularly emphasized by the specific population groups. In Table 3**,** we provided the representative quotes for each population comparison group.

**Table 3:** Representative quotes for population differences among the groups who experienced cancer health disparities.

Population differences	Representative quotes
Race and ethnicity	“Sun exposure, we don’t really hear too much about that, especially for African Americans, you think ‘oh, we don’t really need [to know about the risks of] the sun exposure,’ but we need to [watch sun exposure] just as much as everybody else does.” [Black woman from an urban county; aged 29 y] “The Latino community, generally speaking, when parents bring their daughters to their appointments and the doctor starts talking about the HPV [human papillomavirus] vaccination, the doctors also start talking about if the teen’s been sexually active and that doesn’t click so well...perhaps there is a deep cultural and religious background, but many moms are like ‘I don’t see the need for that vaccination, because my daughter is not sexually active.’” [Latina woman from an urban county; aged 50 y] “Some people really don't go to the doctor unless they know for a fact, something is wrong with them. And, sometimes I think in the Black community, there is a lack of trust with health care providers, and I think that sometimes there’s a lot of fear. It’s more out of sight, out of mind. If I don't have anybody telling me that there’s nothing wrong with me, then I don't have anything to worry about. So, kind of that type of thing is a cultural thing as well, and people just don’t go to the doctor. It’s just not something that’s done unless you know, something is wrong.” [Black woman from an urban county; aged 30 y]
Rural and urban residency	“Transportation and the fact we only have two clinics. If those clinics don’t offer screenings, then they have to go outside the county...And [it’s] not only transportation, it’s...the gas money, eating if you’re gone very long, and all that goes along with that.” [White woman from a rural county; aged 49 y] “I think that there is a lot available now, more now than there used to be. I still feel that a lot of people still travel. They still travel to larger hospitals in the larger cities because they feel like they can get better treatment, better care. I think a lot of it has to do with trust and outcomes and seeing that there’s success in this area, with treating it. People don’t want to risk their lives going through treatment in a small town where they’re just starting this treatment.” [White woman from a rural county; aged 58 y] “We need to have the doctor that will stay for years. I mean when I was young, the doctor that I saw until I was 18 was the doctor that birthed me, and I know that that is so rare. But, there’s just no relational aspects to health care. You’re just a number, and you’re lucky if you can get a doctor that’ll stay for a few years in a small town. So that’s, you know, I think that’s the big thing is just access, and access doesn’t mean 30 minutes away, it means I can walk there.” [White woman from a rural county; aged 55 y]
Gender norms and LGBTQ+^a^ identity	“It was after the surgery, my follow-up, and I was going to get the pathology report from the breast surgeon. My partner came with me, and I don't think they even offered to have her sit down. They didn't ask her if she had any questions. There was just no, you know, they just kind of ignored her, and it was, you know, not a fun time at all. So, I don’t know how prejudiced they were, but we felt it anyway.” [White woman from an urban county; aged 73 y; LGBTQ+ identity] “I mean, my father was very anti-doctor. No matter what I would say to him...he wouldn't go to a doctor. But at the chiropractor, they said ‘hey, I’m not qualified to say this, but it looks like you have cancer, you’re a very sick man and you need to go to the hospital.’ He died six weeks later. I’d hear him say it many times, he felt like it [health care] was only for the rich...if you can figure out how to change that mentality. [White man from an urban county; aged 41 y; LGBTQ+ identity]
Generational differences	“My mom is in her 60s...this morning, she asked me if her camera was capable of videos...I think not knowing how to do it is a barrier, but maybe you can walk them through how you set it up.” [Black woman from an urban county; aged 29 y] “I think within my family the 30 and under group, we're much more open to [genetic] information and talking to each other about it. Whereas my mom...she’s much more, ‘Why are you sending off your DNA, I don’t want to know,’ and I feel there’s a generational difference in sharing information and understanding why it’s important.” [Black woman from an urban county; aged 30 y]

^a^LGBTQ+: lesbian, gay, bisexual, transgender, queer, and other minority.

#### Race and Ethnicity

The participants expressed several concerns unique to Black and Hispanic or Latino communities, which are the largest minority populations in Kentucky. For example, participants described the need for greater understanding of risk factors specific to their communities, such as obesity or risks from sun exposure. Concerns were regularly raised related to the trust in medical facilities or health care providers, grounded in both historical incidents and current experiences. For example, participants mentioned the need for communication strategies to overcome language barriers and cultural differences. Similarly, they desired an increase in health care providers from racial or ethnic minority groups. Participants often connected trust and cultural factors to reduced receipt of screening and treatment, which co-occurs with fear and the tendency to avoid seeking care until something is wrong. In addition, participants indicated that reduced receipt of support or support programs in racial and ethnic minority communities may be connected to stigma around mental health and fear of asking for help.

#### Rural and Urban Residency

In urban areas, participants identified that most of the risk reduction, screening, treatment, and support discussed in the focus groups were available. Although these resources exist for the most part in larger urban areas such as Lexington and Louisville, participants continued to identify challenges, such as cost, transportation, appointment availability, and perceptions of the quality of local facilities, for urban individuals to access the existing cancer care and support programs.

In rural areas, participants described the presence of regional facilities in some of the smaller urban centers in their vicinity, which may have some resources, although some perceived the quality to be lower than the resources in the larger metropolitan urban areas. Most participants with the ability to do so described choosing to seek out care in larger hospitals with specialized cancer expertise, even if it required driving, finding a place to stay, and taking time off work. Participants expressed the disadvantage faced by individuals lacking resources in receiving quality care, alongside experiences with transient health care in rural areas, which often have shortages of health care professionals. Similarly, participants expressed similar concerns for accessing risk reduction resources, such as healthy foods, places to be physically active, and community programs for tobacco use, which may be available in some rural communities but not accessible to all.

#### Gender Norms and LGBTQ+ Identity

The participants who identified as members of the LGBTQ+ community expressed concerns and frustration with gender and sexuality norms in cancer care. Concerns included the involvement of their partner, or lack thereof, by health care providers in their care experiences; the presence of religious messaging or iconography creating fear or discomfort in cancer care; and ways that traditional gender norms were expressed in treatment decision-making by health care providers and other acquaintances. In addition, among the participants who identified as male, we heard an increased reference to cultural norms surrounding traditional masculinity and a hesitancy to seek medical care.

#### Generational Differences

We also heard some specific differences by age group in cancer experiences. Among older individuals, the participants identified low technology awareness and skills as challenges in the receipt of cancer screening and care, particularly for recent increases in telehealth. Similarly, participants expressed a greater hesitancy in certain screening and treatment options among older individuals, such as an increased skepticism and fear around genetic testing. Younger participants described a lack of attention to resources or support programs targeted toward them and the need to increase the diversity of resources to account for their specific needs.

### The COVID-19 Pandemic and Cancer Care

Finally, given that the focus groups were conducted in 2021, the COVID-19 pandemic played an active role in our conversations, particularly regarding its impact on cancer health care experiences. For example, the participants commented that the COVID-19 pandemic had influenced their trust in medical facilities; caused postponed screening and procedures, thereby potentially contributing to worsening late-stage diagnoses or treatment outcomes; and affected their overall future health due to changes in risk reduction and care-seeking behaviors during this time. Participants described a growing fear of hospitals, not wanting to leave their house, worn-out staff, and not allowing guests during appointments as significant impacts of the COVID-19 pandemic on cancer-related health care, including screening and treatment.

## Discussion

### Principal Findings

Through our online focus groups, we were able to capture a wide range of perspectives on needs across the care continuum throughout the state of Kentucky. Although many excellent risk reduction resources exist in Kentucky, the findings suggest that novel approaches are needed to make information accessible to all communities and to use messaging that will not be interpreted as blaming or shaming. A unique opportunity exists to share information on environmental exposures, as participants knew less about such exposures compared to other risk areas. Similarly, screening efforts need to continue to reach individuals in their respective environments (eg, mobile units, at-home screening, and increasing local screening availability) and to include messaging from individuals who engender trust (eg, local leaders, faith communities, local community events, and schools). Continued efforts are also needed to address practical concerns for both screening and treatment. Despite the increase in potential screening opportunities from improved health insurance coverage from the Affordable Care Act, many individuals continued to identify cost and transportation as playing a role in decision-making and a lack of knowledge on the forms of screening or preventive measures that are covered by insurance.

Particularly among our minority group participants, categorized by race, ethnicity, sexual identity, and gender identity, there is a need for enhanced methods to promote positive and understanding communication between patients and health care providers. This is essential for creating safe care spaces that consider how cultural and gender norms influence cancer care. To address the community-specific challenges faced by individuals, messaging and resources specific to these communities are needed to fight stigma and to improve health disparities. In addition, the participants identified treatment navigation as of particular importance for patients with cancer, with generally positive but varying experiences. Finally, the COVID-19 pandemic has shaped a wide variety of cancer care experiences over the past few years, which has affected receipt of care and may continue to have lasting effects on cancer-related behaviors and health decision-making in the future.

### Comparison With Previous Research

Our findings align with and support the expansion of previous findings using qualitative methods in cancer-focused community health needs assessments. For example, our participants reiterated the importance of culturally appropriate educational materials for cancer screening and treatment by race and ethnicity or rural and urban designation that was found in previous studies [3,6]. Our participants additionally suggested considerations for such materials by gender identity, sexual orientation, and age. Similarly, as in past research, our study participants highlighted the importance of the source of information, connecting this source with experiences of stigma, distrust, and discrimination [2]; however, they requested new and additional information in educational materials, such as environmental exposures and survivorship resources specific to their communities. Similarly, our participants described continuing challenges related to accessing cancer care (eg, distance to care, transportation, and cost) that have also been found in existing studies, as well as specific, unique challenges for rural and urban Kentucky communities [2-4,6].

Our work also adds to the currently described data collection processes used in CNAs among NCI-designated cancer centers, which are required to assess and respond to the needs of their catchment areas [28,29]. Recent studies [2,30-32] have described various methods of capturing community needs for catchment area assessments. However, most of these assessments focus on quantitative analysis of community-based surveys or secondary data, and a few describe the explicit inclusion of qualitative data collection. One recent study [2] included qualitative key informant interviews with organizational partners as a part of their assessment; however, to the best of our knowledge, focus group findings with community members for this purpose have not yet been reported. Furthermore, qualitative data have been more widely used in cancer-related needs assessments or studies focused on specific localities [3,6] or population groups, such as rural [33], gender-based [34], immigrant [35], and racial and ethnic minority populations [15,36], rather than for broader assessments of the needs across a statewide catchment area. Our findings support several key areas identified in the previously reported catchment area assessments. For example, several assessments [2,30-32] highlighted similar findings about the importance of social factors related to health care provider interactions, discrimination, and trust, as well as access to care challenges, such as financial, geographic, and insurance-related barriers.

We used an online format to reach the diverse population of Kentucky to capture in-depth community perspectives across a state with wide-ranging geographic and cultural contexts. Using this format allowed us to successfully document the resources and needs across the cancer care continuum domains. Although online focus groups have been possible for decades, the COVID-19 pandemic has compelled researchers and health professionals to adapt to existing restrictions and caused a rapid expansion in the use of this technology for qualitative data collection [37]. Researchers have established that online focus groups meet the previously determined standards of in-person focus groups [38]; however, with the pandemic-stimulated increase in uptake and technology improvements, more work is needed to establish best practices for the online format [37]. As in previous studies, we obtained substantial data from participants who may not otherwise have been able to participate due to barriers associated with in-person focus groups, such as the need for a central meeting location, transportation, and childcare [11,14]. In addition, a few potential participants indicated that technology would pose a challenge for their participation, which aligns with previous findings of satisfaction or even preference for this format [14]. Although many focus group studies seek to recruit homogeneous samples based on defined characteristics [39], online focus groups gave us the ability to broadly recruit participants in 25 different Kentucky counties. This geographic breadth allowed for a broader perspective than is typically obtained for in-person groups within specific communities.

Previous studies have recommended that CNAs include individuals from the most susceptible populations who experience cancer health disparities [2,31,32]. Our study followed this recommendation through our stratified design, strategically oversampling key target groups, including racial and ethnic minority groups, sexual and gender minority groups, and rural Appalachian communities. By intentionally recruiting a wide variety of Kentuckians, we were able to uncover social determinants of health that influence health equity in the state.

### Strengths and Limitations

In this study, we faced several recruitment and technology-based limitations. First, as conducting recruitment during the pandemic limited face-to-face opportunities, we supplemented these efforts with online strategies, such as digital flyers through the KCP and the KCC listservs and ResearchMatch. The REDCap eligibility screener could be completed on a mobile device or computer, which increased potential participation but could have resulted in higher participation among those with increased access to technology. Second, we sought to address potential technology challenges that may arise from conducting the focus groups on Zoom, such as a lack of internet access or low technology literacy. We asked participants about their comfort level during recruitment and provided support to those who expressed discomfort with the online video format to ensure their ability to participate (only n=2, 4% of the 51 participants requested and received this one-on-one assistance). Third, we tried to ensure we captured lay community member perspectives by recruiting those who do not currently hold a position in health care; however, some participants were particularly knowledgeable about health services in their community due to a background in social services or education, which may intersect with health resources.

Finally, while fairly robust for a qualitative study, our sample size may not have allowed us to capture the full range of thoughts on cancer care in Kentucky, and the purposive sampling design is not intended to be transferable to other states or throughout the United States. Considering our statewide focus, our sample was fairly representative of the state of Kentucky’s population demographics. Our study had an oversampling of female participants, which is a common challenge in qualitative research, and despite our sampling design, we had an underrepresentation of Appalachian counties. Although we successfully oversampled Black or African American and LGBTQ+ participants, we did not recruit enough participants in other racial or ethnic groups to explore their perspectives at a more granular level, and nor did we meet our target for Hispanic or Latino individuals. To address this limitation and to allow for participation in Spanish, we are conducting a supplemental needs assessment focused on the Hispanic or Latino population in Kentucky. In addition, we did not perform member checking for the focus group findings; however, we subsequently collected additional data with CNA participants, allowing for further feedback on these results.

### Future Directions

After this study, we combined the focus group findings with our quantitative data to conduct concept mapping [40,41] to prioritize the diverse needs and strategies uncovered in the CNA process. This prioritization and active participation by community members and partners in the process will allow for the steering committee and other organizations focusing on cancer in Kentucky to hone and deliver programs grounded in community recommendations. In addition, our success with conducting online focus groups in a catchment area needs assessment suggests that this method could be an excellent supplement to future CNAs. Cancer centers and researchers interested in broadly capturing the needs in a large, diverse geographic area may benefit from this approach. This method can also be used by cancer centers in partnership with state, tribe, and territorial cancer control programs and coalitions to inform their cancer plans, aligning with efforts to build communities of solution [42].

### Conclusions

Overall, the high-quality data gathered from our online CNA focus groups will inform future studies and cancer prevention and control efforts in Kentucky, as well as CNAs conducted by cancer centers across the country. Using an online format, we uncovered a wide variety of factors affecting Kentuckians across the cancer care continuum, highlighted the role of social determinants of health, and identified health equity issues affecting different population groups. By conducting community-engaged, qualitative data collection, we provided valuable depth of understanding to complement the interpretation of quantitative data and created a platform for ways to develop future programs and research to address cancer incidence and mortality in Kentucky. These findings suggest that the inclusion of online focus groups can be a valuable component of multimethod CNAs to capture cancer-related needs and solutions in large geographic areas and diverse populations. Structuring focus group questions and analysis based on the cancer care continuum domains provides useful data for CNAs and stratifying the focus groups based on similar demographic characteristics facilitates identifying unique needs of specific groups that experience cancer health disparities.

## Appendix

Multimedia Appendix 1 Semistructured focus group discussion guide.

## Abbreviations

CIO: Community Impact Office
CNA: cancer needs assessment
KCC: Kentucky Cancer Consortium
KCP: Kentucky Cancer Program
LGBTQ+: lesbian, gay, bisexual, transgender, queer, and other minority
NCI: National Cancer Institute
REDCap: Research Electronic Data Capture
UKMCC: University of Kentucky Markey Cancer Center

## Data Availability

The datasets generated during or analyzed during this study are available from the corresponding author on reasonable request.
