# Supplementary figures and images for: Capturing Community Perspectives in a Statewide Cancer Needs Assessment: Online Focus Group Study
Source: JMIR Cancer. 2025 Jul 31;11:e63717. doi: 10.2196/63717 (PMC12355136; doi:10.2196/63717)

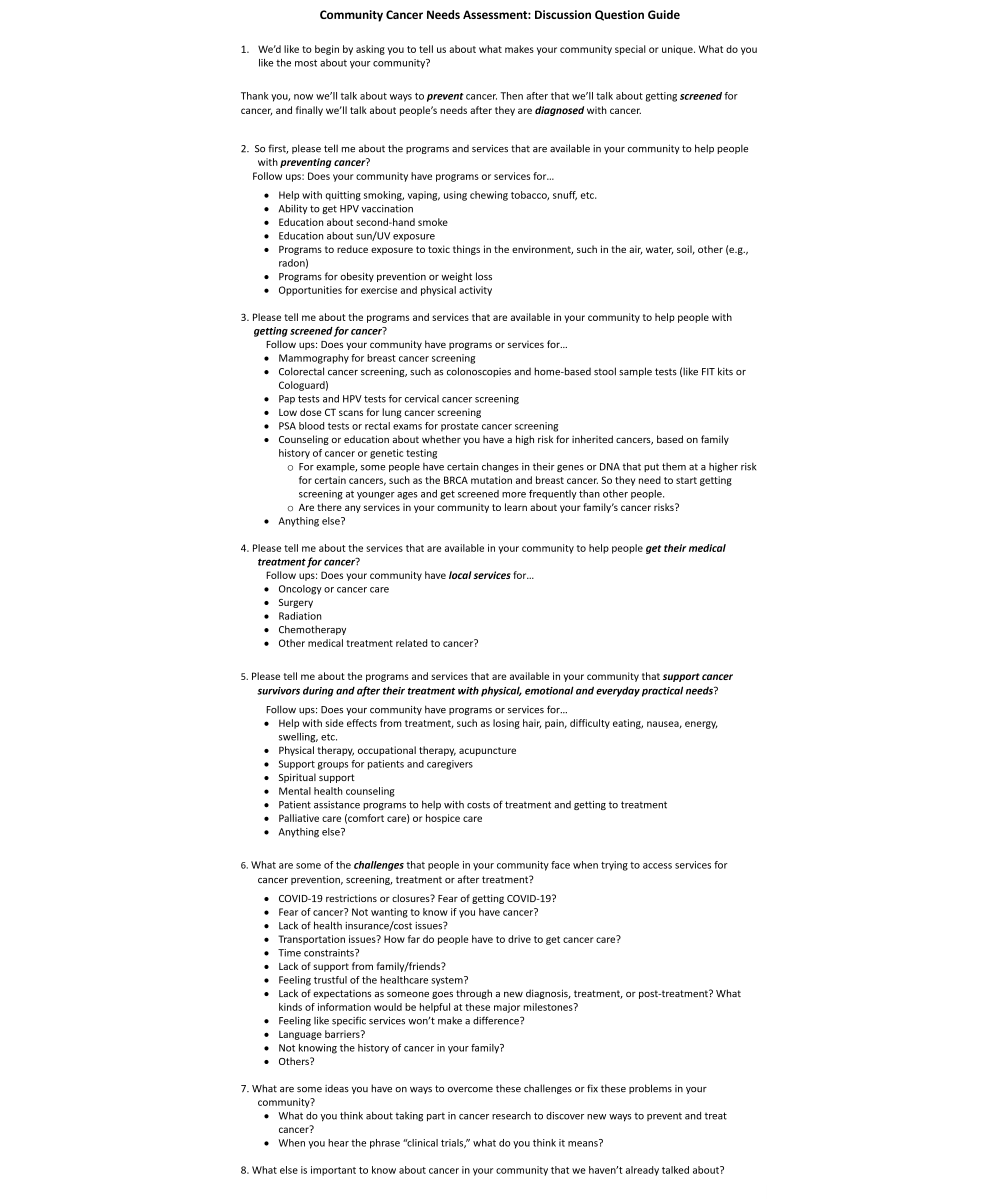

Supplement: Multimedia Appendix 1 [file cancer_v11i1e63717_app1.png]
